# MCPIP1 RNase and Its Multifaceted Role

**DOI:** 10.3390/ijms21197183

**Published:** 2020-09-29

**Authors:** Richard Musson, Weronika Szukała, Jolanta Jura

**Affiliations:** Department of General Biochemistry, Faculty of Biophysics, Biochemistry and Biotechnology, Jagiellonian University, Gronostajowa 7, 30-387 Krakow, Poland; richardmusson@gmail.com (R.M.); weronika.szukala@doctoral.uj.edu.pl (W.S.)

**Keywords:** MCPIP1, RNase, adipogenesis, angiogenesis, differentiation, cancer, skin inflammation

## Abstract

Inflammation is an organism’s physiological response to harmful septic and aseptic stimuli. This process begins locally through the influx of immune system cells to the damaged tissue and the subsequent activation and secretion of inflammatory mediators to restore homeostasis in the organism. Inflammation is regulated at many levels, and one of these levels is post-transcriptional regulation, which controls the half-life of transcripts that encode inflammatory mediators. One of the proteins responsible for controlling the amount of mRNA in a cell is the RNase monocyte chemoattractant protein-induced protein 1 (MCPIP1). The studies conducted so far have shown that MCPIP1 is involved not only in the regulation of inflammation but also in many other physiological and pathological processes. This paper provides a summary of the information on the role of MCPIP1 in adipogenesis, angiogenesis, cell differentiation, cancer, and skin inflammation obtained to date.

## 1. Introduction

### 1.1. Inflammation

Inflammation is a biological response activated by the immune system in response to harmful stimuli such as injury or invasive pathogens. This process forms part of the ‘innate’ immune system because it is a generalised reaction to noxious stimuli and not part of the ‘adaptive’ immune system, which is responsible for the recognition and elimination of specific pathogens by specialised cells [1]. Innate immune cells such as macrophages, fibroblasts, and leukocytes possess pattern recognition receptors (PRRs) either on their cell surfaces or inside the cells.

PRRs detect biomolecules from two main sources: invading pathogens possess pathogen-associated molecular patterns (PAMPs), and damaged cells release damage-associated molecular patterns (DAMPs) [2]. PRRs can be divided into two groups based on their subcellular location: membrane-bound PRRs include Toll-like receptors (TLRs) and C-type lectin receptors (CLRs), and intracellular PRRs include RIG-I-like receptors (RLRs), AIM2-like receptors (ALRs), and NOD-like receptors (NLRs) [3]. The activation of PRRs by PAMPs or DAMPs might activate transcription factors such as NF-κB, AP1, CREB, c/EBP, and IRF to initiate the immune response. Immune cells might then produce mediators that act on target tissues to alter their functional state in response to noxious conditions. For example, TLRs, a well-studied type of PRR that detects invading microbes, produce inflammatory cytokines (e.g., TNF-α, IL-1β, and IL-6) and chemokines (e.g., MCP-1 and CXCL8) that act as mediators in the inflammatory response [4]. These mediators can alter the behaviour of other immune cells, such as by instigating the recruitment of phagocytic neutrophils to sites of infection, or can alter the behaviour of target tissues, such as by promoting vasodilation.

The exact type of inflammatory response, as well as its ultimate effects, varies depending on the specific noxious stimulus: bacterial infections activate different pathways and recruit different immune cells than viral or parasitic infections, and responses to injury and inorganic irritants differ depending on their severity and location in the body. To fully maximize the effectiveness of an appropriate immune response to a noxious stimulus, PRR activation is often the first stage of an inflammatory cascade and allows the triggering of an amplified reaction by a small number of activated cells. When activated by PAMPs and DAMPs, a subset of ALRs and NLRs can assemble into protein complexes called inflammasomes [5]. Inflammasomes catalyse the processing of inactive pro-caspase-1 to active caspase-1, which activates the inflammatory cytokines IL-1β and IL-18 and initiates pyroptosis, a highly inflammatory form of programmed cell death. Inflammasomes are therefore crucial for inflammatory signalling cascades, but their dysregulation is also linked to autoimmune, metabolic, and neurodegenerative diseases.

Inflammation is a critical element of host pathogen defence and wound healing. This process functions as a means to wall off and quarantine affected areas and simultaneously allows immune cells to infiltrate tissues to facilitate pathogen destruction and promote recovery [6]. However, inflammation also exerts marked deleterious effects on cell and tissue function. Obviously, cells and tissues that are inflamed cannot perform their everyday functions as effectively as under normal conditions, which is likely expected because inflammation is a response to unusual and disruptive cell conditions. Despite this fact, as cells begin to adapt their behaviour to their new environments or to restore homeostatic physiological conditions, they might activate metabolic switches or activate stress responses and thereby increase their vulnerability to certain types of diseases [4].

Inflammation is a physiological condition, however, prolonged action of harmful factors or dysregulation of restoring inflammation homeostasis leads to the development of chronic inflammation, which is a pathological condition. In general, inflammation is associated with the innate immune response, while the second, chronic phase, is associated with activation of adaptive immune responses. Innate immune cells (monocytes, neutrophils, natural killer (NK) cells) release proinflammatory cytokines including tumor necrosis factor (TNF), IL-6, IL-12, and type I and II interferons (IFNs). Innate immune cells cooperate with adaptive immune cells (T and B lymphocytes) to orchestrate the nature of inflammation and its consequences. Activated adaptive immune cells (APCs) also produce numerous proinflammatory cytokines including IL-12, IL-6, and type I IFNs and other mediators of inflammation responsible for the proliferation and differentiation of B-lymphocytes and T-lymphocytes after antigen recognition and the activation of effector cells. Among them are; IL-2, IL-4, IL-5, IL-13, IFN-γ, transforming growth factor beta (TGF-β) [2,4,7,8,9].

Chronic inflammation is considered to have an impact on many processes, such as, angiogenesis, adipogenesis, proliferation, differentiation and as a consequence, it contributes to many diseases, including cancer, metabolic syndrome, arthritis, asthma, chronic obstructive pulmonary disease (COPD), Alzheimer’s disease, chronic kidney disease (CKD), inflammatory bowel disease (IBD), and cardiovascular diseases [10]. A state of chronic inflammation might arise if the original noxious insult is not resolved; for example, some infectious organisms, such as *Mycobacterium tuberculosis*, can resist host defences and remain in the body for a long time, resulting in a persistent inflammatory state [11]. Additionally, exposure to pollutants or particulate irritants as part of daily life can also result in chronic inflammation, and this condition is a reality for many people living in dense, smoggy cities or who work in certain types of industry, such as construction using concrete and granite, which can produce silica dust [12]. The classic sign of autoimmune disorders and cellular/genetic disorders such as familial Mediterranean fever is also chronic inflammation [13].

### 1.2. MCPIP Family

The existence of endogenous proteins that act as anti-inflammatory mediators was hypothesised many decades ago, and sporadic evidence indicates that the implantation of irritant substances stimulates specific protein production [14]. However, no proteins with clear anti-inflammatory activity were identified for a long time, and greater emphasis was placed on vasoactive substances, cytokines, bioactive lipids, and adhesive reactions between individual inflammatory cells [15]. One of the first proteins that was found to exert demonstrable anti-inflammatory effects was vasoactive intestinal polypeptide (VIP), but its roles in inflammation were not recognized until approximately 30 years after its initial discovery [16,17,18].

In 2006, the first member of a new family of anti-inflammatory proteins was discovered: monocyte chemoattractant protein-induced protein 1 (MCPIP1). The name of the protein is due to its activation by monocyte chemoattractant protein-1 (MCP-1), which is known chemoattractant responsible for the migration of monocytes/macrophages and involved in inflammatory processes [19]. Similar to VIP, however, its anti-inflammatory behaviour was not immediately noticed. Instead, MCPIP1 was initially described as a novel transcription factor that induces HEK-293 cell apoptosis [19]. Two years later, the same laboratory published their discovery of three similar proteins, resulting in four members of the MCPIP family: MCPIP1-4, encoded by the genes ZC3H12A-D, respectively [20]. The description of MCPIP1 as a transcription factor appeared to be an error caused in part by the presence of a single CCCH-zinc finger motif. This motif is now appreciated as a key structural feature shared by all four MCPIP proteins and is necessary for the most important function of the MCPIP family: RNA degradation.

**Figure 1:**
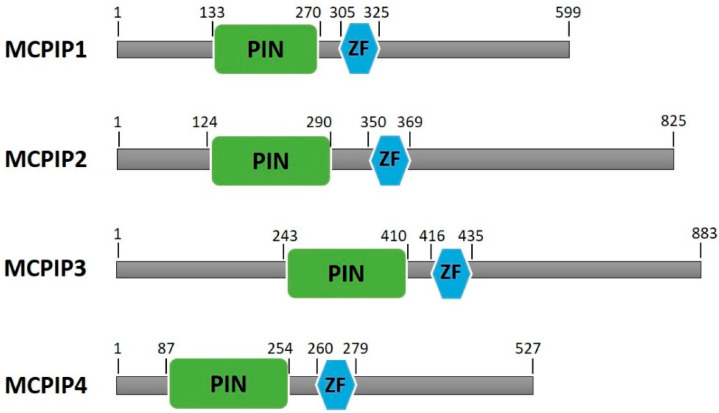
Schematic representation of human MCPIP family members containing the PinT N-terminus domain (PIN) and CCCH-type zinc finger domain (ZF). Based on Lin et al., 2013 [22].

In addition to zinc fingers, all four MCPIP members possess PilT N-terminus (PIN) domains (Figure 1). After the recognition of and binding to target mRNA molecules, the cleavage and regulation of targets is accomplished by PIN domains. The sequence homology of these domains between family members is at least 72% [20,21].

MCPIP1 has been much more extensively studied than the other three family members, although growing evidence suggests that all four proteins engage in regulating inflammatory processes. Given their close sequence homology, it should come as no surprise that they share similar functions.

### 1.3. MCPIP1

MCPIP1 (also known as regnase-1) is encoded by the *ZC3H12A* gene and is composed of 599 amino acids that encode a 66-kDa protein. MCPIP1 is a multifaceted anti-inflammatory protein that plays a critical downregulatory role in the immune inflammatory response through at least two independent mechanisms: destabilising the mRNA transcripts of many cytokines and inhibiting lipopolysaccharide (LPS)- and IL-1β-induced NF-κB signalling [23].

MCPIP1 possesses a well-documented ribonuclease ability, as demonstrated by its targeting of many cytokine mRNAs for direct degradation; specifically, previous studies have shown that MCPIP1 degrades IL-1β, IL-6, IL-8, IL-12p40, and IL-17 mRNA transcripts (Table 1) [24,25,26,27].

The regulation of mRNA transcripts is one of the primary mechanisms through which protein levels are controlled: these molecules can be protected or destroyed to alter the amount of protein being translated under specific circumstances.

Across all organisms, mRNA destabilisation and decay can be performed via a variety of pathways, such as the targeting of conserved AU-rich elements (AREs) and stem-loop structures (SLs) or nonsense-mediated decay (NMD), which prevents the translation of mRNAs. Mino and colleagues discovered that MCPIP1 degrades IL-6 mRNA via the SL structure in the 3′-UTR region, and IL-6 mRNA molecules lacking this sequence were not degraded [30]. More recently, Wilamowski et al. observed that IL-6 is degraded by MCPIP1 in a progressive manner: after SL is cleaved, multiple shorter single-stranded RNA (ssRNA) molecules are generated, and these molecules are then further degraded by MCPIP1. Interestingly, these researchers also found that a 6-nt RNA molecule was bound but not degraded, possibly because it is too short to reach the catalytic site [31].

**Figure 2:**
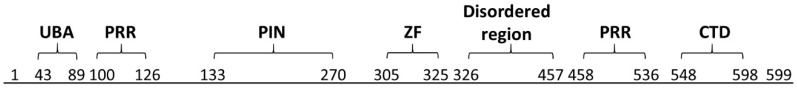
Domains of MCPIP1. Ubiquitin-associated domain (UBA) 43–89; proline-rich region (PRR) 100–126 and 458–536; PilT N-terminus nuclease domain (PIN) 133–270; zinc-finger motif (ZF) 305–325; disordered region 326–457; and C-terminal conserved domain (CTD) 545–598. Based on Wilamowski et al., 2018 [31].

The critical RNase capability of MCPIP1 is due to its PIN domain (Figure 2) [31]. PIN domains, which are a common motif found in both prokaryotic and eukaryotic nucleases, are primarily responsible for binding to and degrading RNA molecules and also play roles in the *bacterial* stress response and pathogenesis [32]. The importance of the PIN domain was demonstrated by Matsushita and colleagues, who used site-directed mutagenesis to alter one amino acid (D141N) and found that this alteration completely abolished the RNase function [25].

Within the PIN domain, four aspartate residues act in coordination with a single magnesium ion to assemble a catalytic cleft, which constitutes the active site of MCPIP1. This positively charged loop sequence might be responsible for binding to RNA by specifically attracting the negatively charged phosphate groups of oligonucleotide backbones [33].

**Figure 3:**
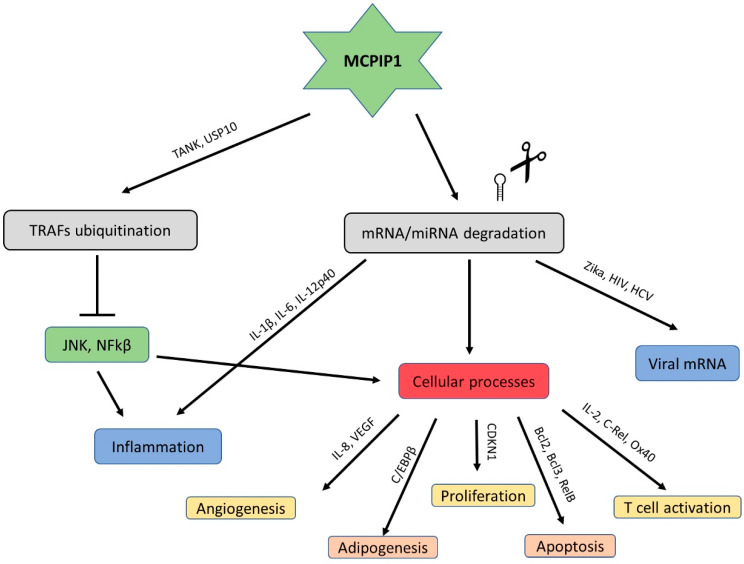
Schematic modes of action of MCPIP1.

The USP10-dependent deubiquitination of NEMO and TRAF6 proteins is another strategy through which MCPIP1 can regulate inflammation and the immune response [34]. It results in the negative regulation of the transcription factors c-Jun N-terminal kinase (JNK) and NF-κB [35]. JNK and NF-κB signalling mediate many cellular responses, including infections, inflammation, and apoptosis, through the transcriptional activation of several cytokine genes (Figure 3) [36].

MCPIP1 also possesses a CCCH zinc finger domain close to the C-terminal region of the PIN domain (Figure 2). Zinc finger domains are commonly regarded as DNA-binding, but CCCH-type zinc fingers, among others, are involved in binding to RNA molecules and regulating their metabolism [37]. CCCH zinc finger proteins are particularly associated with immune responses and play roles in antiviral innate immunity, the production of cytokines, immune cell activation, immune homeostasis, and the regulation of cell differentiation and cancer cell growth. For example, Roqiun-1, another CCCH-type zinc finger protein, causes a lupus-like autoimmune disease in mice when mutated [38].

**Figure 4:**
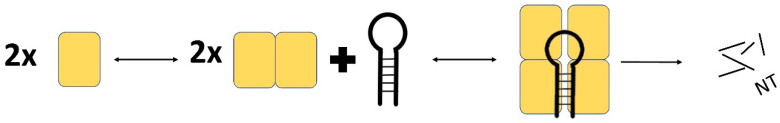
Schematic presentation of the ternary complex model of MCPIP1. Based on the size exclusion chromatography results Wilamowski et al. proposed a sequential binding model: oligo  +  MCPIP1_dimer_  +  MCPIP1_dimer_ ⇄ oligo-MCPIP1_dimer_  +  MCPIP1_dimer_ ⇄ oligo-MCPIP1_tetramer_ (MCPIP1 marked as a yelow box). MCPIP1 degrades RNA molecules as a tetramer [31].

MCPIP1 appears to undergo homooligomerisation during interaction with RNA substrates (Figure 4). Size-exclusion chromatography revealed that a dimeric form of MCPIP1 appears to be the most common under native conditions, although tetrameric and monomeric fractions might also be present [31]. This homooligomerisation occurs through the proline-rich C-terminal domain [21]. Mutations that prevent oligomerisation also abolish RNase activity, which indicates that oligomerisation is crucial for MCPIP1 enzyme function [39]. Other RNases and RNase domains also function in an oligomerised state; for example, RNase A oligomerises via a proline-dependent arm exchange mechanism [40].

The C-terminal region appears to be crucial for another function of MCPIP1: MCPIP1 plays a broad role in suppressing microRNA (miRNA) activity and biogenesis [21]. miRNAs are small non-coding RNA molecules that perform RNA silencing and post-transcriptional regulation of gene expression. MCPIP1 RNase blocks Dicer processing and prevents pre-miRNAs from developing into mature miRNAs. MCPIP1 might recognize and degrade pre-miRNAs in a similar way to mRNAs by targeting terminal loop structures and cleaving RNA with the PIN domain [21]. Both human and viral pre-miRNAs are cleaved by MCPIP1. However, some viral miRNAs might be able to resist this effect and repress MCPIP1 expression, as evidenced by Happel and colleagues, who studied the relationship between MCPIP1 and miRNAs in Kaposi’s sarcoma-associated herpesvirus (KSHV) [41].

## 2. Broad Roles of MCPIP1 in the Immune System

The crucial role that MCPIP1 plays in the regulation of inflammation can be clearly demonstrated by knockout mice. *ZC3H12A-/-* mice exhibit a severely altered phenotype characterized by drastic immune system dysregulation: these mice suffer growth retardation and spontaneous death usually within 12 weeks [25]. In these mice, immune cells are overactivated and hyperinvading, resulting in splenomegaly, lymphadenopathy, anaemia, and hyperimmunoglobulinemia. These deviations from normal physiology do not appear until after birth; newborn *ZC3H12A-/-* mice do not exhibit many obvious differences from their wild-type littermates. After approximately 3 weeks, however, the spleen sizes are notably larger, and leukocyte infiltration into the interstitial spaces of the lungs can be observed. In addition, proinflammatory cytokines, such as IL-6 and IL-1β, exhibit markedly higher expression in MCPIP1-deficient mice.

Interestingly, treatment with antibiotics improves the lifespan and ameliorates hyperinflammatory syndrome in knockout mice [42]. This finding might be explained by host-microbiota interactions: it has been hypothesized that commensal microbiota in mucosal surfaces and the digestive system can elicit TLR signalling and thereby contribute to the development of some autoimmune diseases such as IBD [43]. Therefore, this finding might indicate another function of MCPIP1 within the immune system: attenuation of baseline TLR signalling by resident microorganisms.

The role of MCPIP1 as a broad negative regulator of inflammation is also evidenced by the stimuli that can induce its expression. *ZC3H12A* expression is induced by a number of proinflammatory factors, although the strongest inducers might depend on the cell type. For example, in human hepatoma HepG2 cells, IL-1β is an effective stimulant, whereas in promonocytic U937 cells, TNF is the strongest inducer of MCPIP1 [24]. The expression of the *ZC3H12A* gene is also rapidly induced by many factors that are present during infectious attack by pathogens. MCPIP1 expression is increased during viral, bacterial, and fungal infections [44]. LPS, the main outer membrane component of gram-negative bacteria, and the mycobacterium tuberculosis 38-kDa antigen both trigger TLR signalling and subsequently increase MCPIP1 expression [45,46].

However, MCPIP1 appears to exert an overall relaxing effect on immune cell activation. Conditional T-cell MCPIP1-knockout mice exhibit greatly increased rates of T-cell activation, which leads to the conclusion that MCPIP1 plays a role in suppressing the activation of immune cells. MCPIP1 degrades the mRNAs of the genes c-Rel, Ox40, and IL-2, which are responsible for the activation of T cells [47]. MCPIP1 works cooperatively with Roquin to suppress the differentiation of proinflammatory T helper 17 (Th17) cells [48]. Moreover, MCPIP1 negatively regulates group 2 innate lymphoid cells (ILC2s) functions, which are a critical innate source of type 2 cytokines in allergic inflammation. Matsushita et al. discovered that IκB kinase (IKK) complex–mediated MCPIP1 degradation is essential for IL-33– and IL-25–induced ILC2 activation [49].

Despite this effect of limiting immune cell activation, MCPIP1 might play a role in defending the host from foreign nucleic acids such as viruses. Qian and colleagues demonstrated that MCPIP1 can distinguish between mRNAs from exogenously transfected plasmids and those from the host genome and selectively degrade foreign transcripts. Additionally, these researchers showed that the induction of MCPIP1 can restrict Zika virus infection and thereby significantly decrease the viral RNA levels [22,50,51,52]. MCPIP1 also potently inhibits other viral infections, including HIV-1 and Hepatitis C Virus (HCV) infection [42,44].

### 2.1. MCPIP1 Regulation

The undisputed main role of MCPIP1 is to suppress the inflammatory immune response, which is crucial for preventing a state of chronic inflammation. However, the strong activation of MCPIP1 during pathogen infection would create a favourable environment for the pathogen to invade and replicate. Therefore, pathways need to be in place for the regulation of MCPIP1 and prevent its interference in the immune system’s fight against pathogens. Uehata and colleagues discovered that T-cell receptor activation triggers MCPIP1 degradation by the protease Malt1 [47]. Similarly, the IKK complex ubiquitinates and degrades MCPIP1 after stimulation via TLRs or IL-1β [53].

Another mode of MCPIP1 regulation is the self-degradation of its own transcript [25]. This behaviour establishes a feedback loop to ensure that the immune response is never significantly handicapped by high levels of MCPIP1 expression. Similar to the mechanism through which MCPIP1 degrades many mRNA transcripts, it is thought that this self-regulatory activity is also stimulated by the targeted nucleolytic cleavage of the stem-loop structure contained within the 3′-UTR of MCPIP1 mRNA; luciferase assays with truncated 3′-UTR sequences provide evidence to support this finding [53]. However, another study found that MCPIP1 mRNA without this 3′-UTR sequence is also degraded by MCPIP1 protein [50]. Clearly, further studies are needed to precisely identify the mechanism through which this feedback loop is established.

### 2.2. Roles of MCPIP1 in the Regulation of Cellular and Bodily Processes

MCPIP1 does not exclusively play important roles in immune function. This factor is expressed in many different cell types and tissues throughout the body. Moreover, MCPIP1 is most highly expressed in leukocytes but is also present in the heart, placenta, spleen, liver, kidney, and lung [25]. This RNase is also involved in many different processes and has subsequently been implicated in many diseases. Many of these involvements are caused indirectly by molecules that regulate or are regulated by MCPIP1. For example, MCP-1, IL-1β, IL-6, and TNFα are involved in different diseases, including rheumatoid arthritis [54], scleroderma [55], COPD [56], diabetes [57], obesity [58], and cancer [59], among many others.

A growing body of evidence shows that MCPIP1 regulates the differentiation and proliferation of many different cell types, and this effect might be related to the MCPIP1-induced increase in reactive oxygen species (ROS), which leads to endoplasmic reticulum stress. For example, Wang and colleagues demonstrated that the forced expression of MCPIP1 induces monocytes into osteoclast precursors and that this effect is accompanied by increased ROS production via the MCPIP1-mediated upregulation of p47PHOX [60]. Similarly, the blockage of MCPIP1 translation with small interfering RNA (siRNA) can result in less ROS production after cholesterol treatment and thereby lower DNA damage [61]. In contrast, MCPIP1 overexpression suppresses the formation of stress granules (SGs) when cells are exposed to arsenite-induced oxidative stress [62]. Although whether MCPIP1 increases or decreases cellular stress levels is currently unclear, the links to cell differentiation are abundant: neural progenitor differentiation to glial cells [63], angiogenic tube formation [64], and adipogenesis induction in 3T3-L1 preadipocytes [65] are all processes regulated by MCPIP1.

### 2.3. Adipogenesis

**Figure 5:**
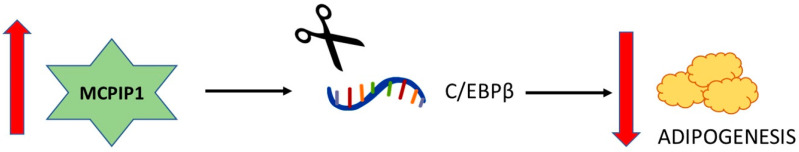
Schematic relationship between the level of MCPIP1 and adipogenesis. A. The overexpression of MCPIP1 decreases the C/EBPβ mRNA level and impairs adipogenesis.

In fact, adipogenesis is one of the most well-studied aspects of the non-immune functions of MCPIP1 (Figure 5). Adipogenesis is regulated by a set of transcription factors, including CCAAT/enhancer-binding protein (C/EBP) β, C/EBPδ, C/EBPα and peroxisome proliferator-activated receptor γ (PPARγ) [66]. Although Younce et al. (2009) observed increased adipogenesis after MCPIP1 overexpression [65], later studies observed an opposite effect of MCPIP1 on adipogenesis. In 2014, Lipert and colleagues demonstrated that MCPIP1 impairs adipogenesis: the overexpression of MCPIP1 decreases the C/EBPβ and PPARγ mRNA levels, whereas the silencing of MCPIP1 increases the expression of these mRNAs [67]. Later studies showed that the MCPIP1 level is decreased in the adipose tissue of obese subjects and that MCPIP1 downregulates genes encoding proteins involved in carbohydrate and lipid metabolism while upregulating genes involved in cellular assembly and movement [68].

### 2.4. Angiogenesis

MCPIP1 has also been linked to angiogenesis. Prior to the discovery of MCPIP1, its inducer MCP-1 was associated with angiogenesis: the direct application of MCP-1 to culture media of human aortic endothelial cells (HAECs) results in upregulated expression of hypoxia-inducible factor 1α (HIF-1α) and vascular endothelial growth factor-A165 (VEGF-A165) [69]. Later studies have shown that these proangiogenic effects are instead most likely caused by MCPIP1 rather than MCP-1. The siRNA silencing of MCPIP1 prevents the occurrence of any angiogenic response; the pro-angiogenic cadherin genes *Cdh12* and *Cdh19* are upregulated by MCPIP1 [64]. The angiogenic capacity of MCPIP1, along with its ability to enhance the cardiac differentiation of mesenchymal stem cells (MSCs), might make MCPIP1 a therapeutic target in the myocardial repair and regeneration of ischaemic tissues [70]. However, more recent studies performed using the clear cell renal cell carcinoma (ccRCC) cell line Caki-1 (metastatic) showed the anti-angiogenic effect of MCPIP1. Caki-1 cells overexpressing MCPIP1 exhibited decreased levels of HIFs, glucose transporter 1 (GLUT1), VEGFA, and IL-6 [71].

## 3. Tumor Immune Response

Furthermore, in vivo studies revealed MCPIP1 as a key player in the vascularization process during tumorigenesis [72]. Low protein levels of MCPIP1 are also correlated with renal cancer progression, as manifested by increased proliferation and tumour outgrowth [72]. The RNA sequencing of Caki-1 ccRCC cells overexpressing MCPIP1 revealed that many downregulated genes are involved in protein folding, cell cycle regulation, hypoxia response, and cell signalling, and the viability of MCPIP1-overexpressing cells was reduced [73]. Other cancers show similar relationships with MCPIP1 expression. Low MCPIP1 expression has been observed in primary neuroblastoma tumours; enforced expression of MCPIP1 in neuroblastoma BE(2)-C cells causes significant decreases in cell viability and proliferation [74]. This effect might be caused by decreased choline transport and the cleavage of miRNA miR-3613-3p [75]. In contrast, the degradation of miR-200 family members by MCPIP1 is associated with pancreatic adenocarcinomas [76]. It is therefore possible that the broad anti-miRNA activity of MCPIP1 might predispose individuals to some cancer types but protect against others. A low level of MCPIP1 is also a signature of breast cancer. Lu et al. proved that MCPIP1 suppressed the growth of breast cancer cells in vivo by inhibiting cell proliferation and at the same time inducing apoptosis by selectively enhancing mRNA decay of antiapoptotic gene transcripts, including Bcl2, RelB, Birc3 and Bcl3 [77]. More recent studies performed using CRISPR-Cas9 mutagenesis screening of metabolism associated factors, showed that MCPIP1-deficient CD8-positive T cells are reprogrammed to long-lived effector cells with extensive accumulation, better persistence and robust effector function in tumours [78]. These results show new avenues to reprogramming T cell state and metabolism in cancer immunity and immunotherapy.

## 4. Role of MCPIP1 in the Skin

In the past few years, a new and exciting branch of study into MCPIP1 has emerged: its activity in the skin. Dobosz and colleagues were the first to recognize that MCPIP1 plays a broad and crucial role in the epithelium by regulating inflammation and maintaining critical skin homeostasis [26]. Epithelial tissue makes up the body’s barrier to the external environment and is thus the first line of defence against microbial pathogens. Epithelial cells fight infection by recognizing PAMPs, initiating immune responses, and secreting antimicrobial proteins and inflammatory cytokines [79]. The main cytokine necessary to perform this task is IL-8 (aka CXCL8), which induces the chemotaxis of neutrophils to affected tissues and promotes angiogenesis [80]. Similar to many other cytokines, IL-8 needs to be regulated because its uncontrolled expression can cause severe symptoms: IL-8 dysregulation has been associated with diseases of epithelial irritation, such as cystic fibrosis and asthma [81,82]. Dobosz and colleagues found that MCPIP1 expression in epithelial cells is higher than that in myeloid cells and that MCPIP1 degrades IL-8 post-transcriptionally, most likely by targeting stem-loop structures on mRNA transcripts [26].

At least two more epithelial inflammatory cytokines are regulated by MCPIP1: IL-36 and IL-17. The dysregulation of IL-36 has been implicated in the pathology of psoriatic disorders; several missense mutations are strongly associated with generalized pustular psoriasis [83]. MCPIP1 is induced by IL-36 and appears to suppress IL-36 signalling by limiting its autostimulatory loop formation, a behaviour that IL-36 shares with members of the IL-1 family [84]. IL-17 family members are important inflammatory responders to stress, injury, and pathogens and are produced by T cells and innate immune cells [85]. Similar to the results obtained with IL-36, IL-17 dysregulation has been implicated in skin conditions, and antibody drugs targeting IL-17 have shown encouraging success in psoriasis treatment [86]. MCPIP1 is induced by IL-17A dependent on STAT3 phosphorylation [87] and appears to inhibit IL-17A and IL-17C signalling because *Zc3h12a−*/− keratinocytes exhibit increased inflammatory responsiveness to IL-17A and IL-17C stimulation [88].

In vivo studies have also demonstrated the crucial roles that MCPIP1 plays in the skin. Studies have shown that MCPIP1 is predominantly localized in the differentiated suprabasal layers in the normal human epidermis [87]. Further studies have shown that MCPIP1 is both transcriptionally and translationally activated in the human psoriatic epidermis [87,88]. Heterozygous *Zc3h12a*+/− mice and (keratin-5) keratinocyte-specific MCPIP1-knockout mice both display increased sensitivity to imiquimod, a TLR-activating skin abrasion chemical [84,88]. This increased sensitivity is manifested by enhanced erythema, epidermal thickening, skin scaling, and ear swelling [88]. Furthermore, Takaishi et al. demonstrated the involvement of IL-36R signalling in the development of the psoriasis-like phenotype [84].

Most recently, Konieczny and colleagues generated keratin-14-specific MCPIP1-knockout mice and provided an extensive physiological characterization [89]. Although newborn and young MCPIP1^eKO^ mice appear phenotypically normal, abnormalities begin to appear at approximately 4 months of age, and chronic wounds and hair loss develop gradually. At approximately 6 months, a significantly reduced body weight (approximately 13%), splenomegaly, and enlarged lymph nodes can be observed. The neutrophil, eosinophil, monocyte, and macrophage numbers in these mice are higher than those in control mice, even though the proportions of B and T lymphocytes among splenocytes are decreased in the knockout mice.

One crucial aspect necessary for the formation of an intact epidermal barrier is that keratinocytes are very tightly regulated in a balance between proliferation and differentiation. This multistage process is mainly triggered by extracellular calcium [90]. Konieczny et al. decided to further investigate the effect that MCPIP1 might play in keratinocyte differentiation and proliferation [89]. First, during the in vitro Ca2+-induced differentiation of normal human keratinocytes, MCPIP1 is upregulated concurrently with keratin 10. To determine the keratinocyte transcriptomes of MCPIP1^eKO^ mice, RNA sequencing was performed. The results revealed that the keratin family of genes (*Krt6b*, *Krt16*, and *Krt23*) and small proline-rich protein 2 (*Sprr2d/e/h*) are upregulated, which indicated that MCPIP1 normally suppresses these genes. These two families are involved in keratinocyte differentiation and have been implicated in innate immunity and inflammation regulation [91,92]. In addition, matrix metallopeptidase 9 (*Mmp9*) is upregulated in these mice. MMP9, similar to other metallopeptidases, is involved in the breakdown of the extracellular matrix and in the migration of keratinocytes [93] but has also been implicated in certain inflammatory diseases, such as psoriasis [94].

## 5. Conclusions

Based on the experimental results obtained to date, MCPIP1 RNase participates in many cellular processes, including both physiological and pathological processes, and its activity depends on the process and the cell type. Nevertheless, in each of these processes, MCPIP1 acts as a regulator of the stability of RNA molecules, such as miRNAs, mRNAs and possibly long non-coding RNAs (lncRNAs). Clearly, MCPIP1 is crucial for the proper functioning of various types of cells. Understanding the precise implications of its action could have profound implications for future research in therapies for diseases, such as psoriasis and metabolic syndrome.

## Figures and Tables

**Table 1:** mRNA of cytokines and chemokines degraded by MCPIP1 in various types of cells.

mRNA	Cells	References
IL12p40	Macrophages	[25]
IL6	Macrophages, cardiomyocytes	[25,28]
IL1β	Macrophages	[24]
IL17RA, IL17RC	Lymphocytes, fibroblasts	[27]
IL8	Epithelial cells	[26]
IL2	T cells	[29]
Cxcl1, Cxcl2, Cxcl3	-	[30]
